# Supplementary figures and images for: The Potential of Soluble Proteins in High-Moisture Soy Protein–Gluten Extrudates Preparation
Source: Polymers (Basel). 2023 Dec 12;15(24):4686. doi: 10.3390/polym15244686 (PMC10748057; doi:10.3390/polym15244686)

## Supplementary data

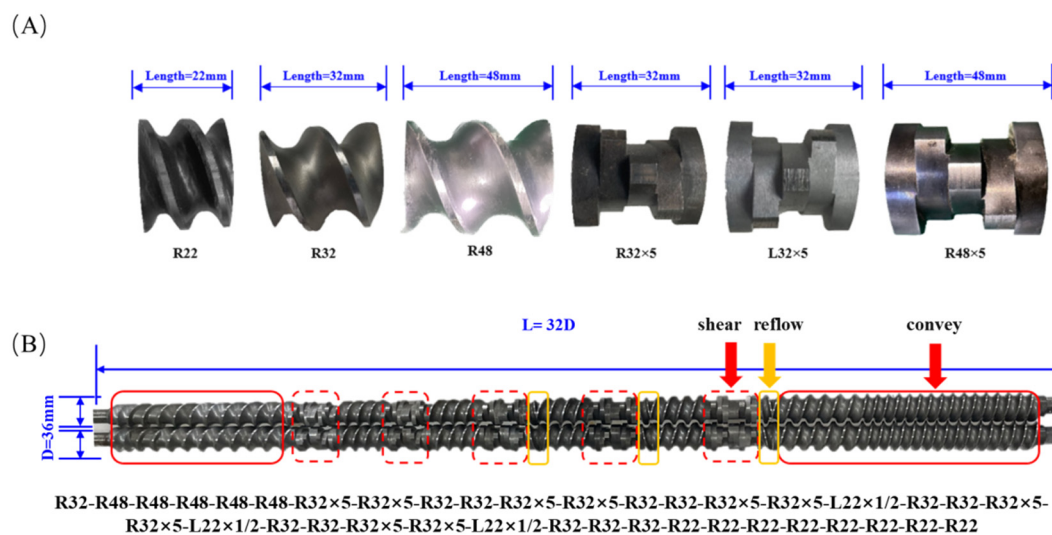

**Figure S1.** Screw elements and design of the screw configuration.

Supplement: Supplementary file 1 [file polymers-15-04686-s001.zip › polymers-2736734-supplementary.pdf]
